# Enhancement of Two-Photon Absorption in Boron-Dipyrromethene (BODIPY) Derivatives

**DOI:** 10.3390/molecules27092849

**Published:** 2022-04-29

**Authors:** Guanzheng Song, Zhongguo Li, Yanbing Han, Jidong Jia, Wenfa Zhou, Xueru Zhang, Yuxiao Wang, Yinglin Song

**Affiliations:** 1 Department of Physics, Harbin Institute of Technology, Harbin 150001, China; gzsong0815@163.com (G.S.); hanyanbing1990@126.com (Y.H.); jjd1130049921@163.com (J.J.); 15862367509@163.com (W.Z.); wangyx@hit.edu.cn (Y.W.); 2 School of Electronic and Information Engineering, Changshu Institute of Technology, Changshu 215500, China; zgli@cslg.edu.cn

**Keywords:** azaBODIPYs, nonlinear optics, two-photon absorption, optical limiting

## Abstract

The linear and nonlinear optical properties of two BODIPY derivatives, 1,7-Diphenyl-3,5-bis(9,9-dimethyl-9H-fluoren-2-yl)-boron-diuoride-azadipyrromethene (ZL-61) and 1,7-Diphenyl-3,5-bis(4-(1,2,2-triphenylvinyl)phenyl)-boron-diuoride-azadipyrromethene (ZL-22), were comprehensively investigated based on experimental and theoretical studies. It was found that both compounds show a strong two-photon absorption response in the near-infrared regime, and the two-photon-absorption cross-section values of ZL-61 and ZL-22 were determined to be 8321 GM and 1864 GM at 800 nm, respectively. The improvement of the two-photon absorption cross section in ZL-61 was attributed to the enhancement of the donor group, which was confirmed by transient absorption measurements and DFT calculation. Our results indicate that these BODIPY derivatives are a promising candidate for optical limiting and two-photon imaging applications.

## 1. Introduction

Organic nonlinear optical (NLO) materials have attracted considerable research attention in the last two decades due to their potential applications in optical power limiting, bio-imaging, optical switching, etc. Among various organic NLO compounds, boron-dipyrromethenes (BODIPYs) derivatives are an intriguing class of chromophores, which is well-known for its excellent fluorescence, good photostability, and structure tunability [1]. Therefore, BODIPY has been widely used in various fields such as photodynamic therapy [2], fluorescent probe [3], cell imaging [4], etc. Aza-BODIPYs, which is a structural analog of BODIPY, have also received a lot of research interest. The major structural difference between aza-BODIPYs and BODIPYs is at the meso-position of aza-BODIPYs, with a nitrogen atom substituting a carbon atom. Due to the highly conjugated structure of BODIPY derivatives, it is possible to fine tune the photo-physical properties of BODIPY by attaching strong electron-donating groups [5] to make the structure rigid [6] or by extending the conjugate length of the π-conjugate system [7].

It is well-known that the chromophores with a large π-conjugated structure are ideal for nonlinear optical applications such as two-photon-induced fluorescence and optical limiting [8]. Therefore, the investigation of the NLO response of BODIPY derivatives attracts great interest. However, previous reports show that the two-photon absorption cross section of BODIPY dye is moderate in the telecommunication band (~500 GM at 1550 nm) but rather low in the visible to near-infrared region (<100 GM) [9,10,11,12]. As a result, a comprehensive investigation of the structure–property relationship of BODIPY derivatives is of great importance for improving the NLO response of BODIPY in the near-infrared regime.

In this work, we designed and synthesized two aza-BODIPY compounds, named ZL-22 and ZL-61, with different electron-donating groups connecting to the BODIPY-core to modulate the optical properties. The TPA cross sections and excited-state dynamics of these aza-BODIPY compounds were systematically studied by femtosecond Z-scan technique and transient absorption measurements. The results of linear and nonlinear optical responses were analyzed via density functional theory (DFT) methods. Our results demonstrate that the TPA cross section of BIDOPY can be significantly enhanced, with a maximum value of 8321 GM at 800 nm.

## 2. Materials and Methods

### 2.1. Synthesis

**Scheme 1:**
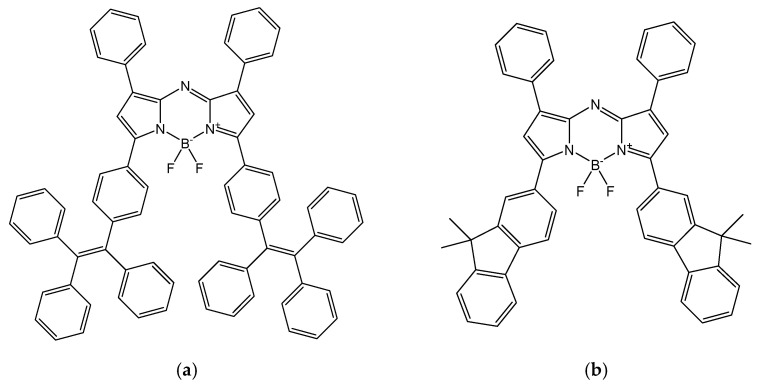
The final synthetic chemical structure of ZL-22 (**a**) and ZL-61 (**b**).

The chemical structure of ZL-22 and ZL-61 both have diphenyl at the 1,7-position. However, at the 3,5-position, ZL-22 is connected with bis4-(1,2,2-triphenylvinyl)phenyl and ZL-61 is connected with bis(9,9-dimethyl-9H-fluoren-2-yl) (Scheme 1). The synthetic procedure of the ZL-61 and ZL-22 compounds can be found in previous literature 14. 

### 2.2. Nonlinear Optical Measurements and Ultrafast Optical Spectroscopy

The nonlinear absorption properties of ZL-22 and ZL-61 compounds were measured by the open-aperture Z-scan technique. The laser source is an Optical Parametric Amplifier (ORPHEUS, Light Conversion) pumped by a femtosecond fiber Yb:KGW laser (PHAROS-SP, Light Conversion), which delivers 190 fs pulses at a 10 Hz repetition rate. The experimental details of Z-scan measurements can be found in our previous report [13]. The concentration used in Z-scan measurements was 2 mg/mL and the sample compounds were contained in 2 mm quartz cuvettes. The excitation wavelength in Z-scan measurements were 800 and 850 nm. The ultrafast excited-state dynamics of the two compounds were investigated via femtosecond transient absorption (TA) spectroscopy measurements. The experimental system of our TA measurements has also described in [13]. In brief, the laser source of the TA spectroscopy measurement is the same as the Z-scan system. The pump pulse is generated by the OPA excited with the Yb:KGW fiber laser. The pump light at 650 nm is operating at 6 KHz with a pulse duration of 190 fs. The changes in absorption upon photoexcitation are probed with a white-light continuum probe pulse (770–1100 nm) that is generated by focusing a part of fundamental 1030 nm light on a sapphire crystal. The pump power was kept below 12 mw to keep the measurement in the weak excitation regime. TA data were acquired by subtracting the probe signal with, and without, the pump beam. All compounds were measured in a quartz cuvette with a 2 mm path length.

### 2.3. Quantum Chemical Calculations

The density functional theory (DFT) was performed to optimize the geometries of compounds, based on the hybrid B3LYP/6-31G(d) level using the Gaussian 09 software package. No imaginary frequencies were observed based on frequency analysis, verifying that the optimized geometries are minimum and stable.

## 3. Results and Discussion

### 3.1. Linear Absorption and Emission

**Figure 1:**
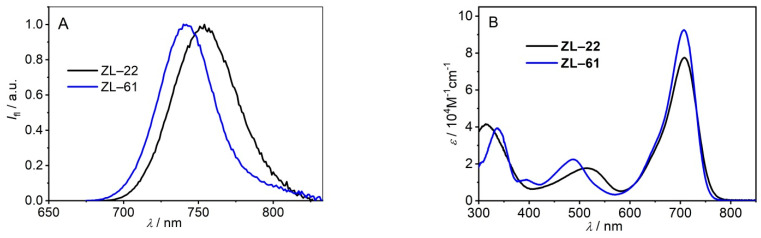
Fluorescence spectra (**A**) and UV-vis absorption spectra (**B**) of the compounds ZL-22 and ZL-61 [14].

The linear absorption spectra of ZL-61 and ZL-22 dissolved in solvent toluene at a concentration of 4 × 10^−6^ M are shown in Figure 1B. According to Zhu’s report [14], the aza-BODIPY dyes, which substitute at the 3,5-positions (which 5a and 5c corresponding to ZL-22 and ZL-61, respectively), have a better electronic conjugated effect due to their higher value of linear absorption. It is found that ZL-22 and ZL-61 have similar UV–Vis spectra with a peak at 708 nm, which is close to near-infrared regions. The strong absorption bands between 600–800 and 400–550 nm can be attributed to the S0→S1 and S0→S2 transitions, respectively. From the fluorescence spectra of the dyes (Figure 1A), which present the mirror image of the S0→S1 absorption bands, the emission peaks corresponding to 754 and 742 nm can be observed, respectively. The linear absorption and emission properties of the ZL-22 and ZL-61 compounds are summarized in Table 1.

### 3.2. Femtosecond Z-Scan Technique and Two-Photon Absorption Cross-Section Calculation

**Figure 2:**
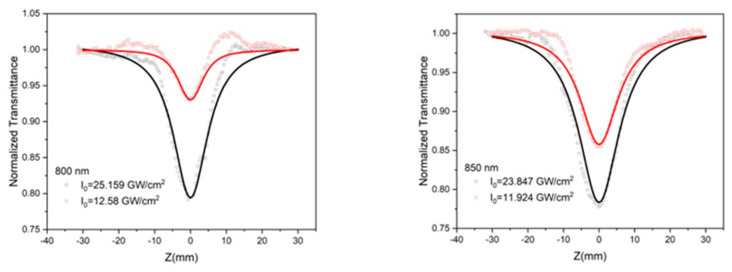
Femtosecond OA Z-scan experiments of ZL-22 at 800 nm and 850 nm. The dots represent experimental data and the lines represent theoretical fitted curves.

**Figure 3:**
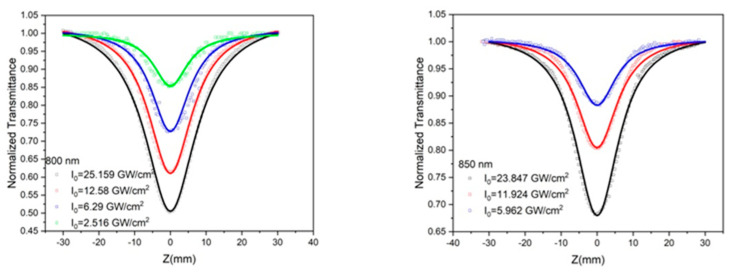
Femtosecond OA Z-scan experiments of ZL-61 at 800 nm and 850 nm. The dots represent experimental data and the lines represent theoretical fitted curves.

The femtosecond open-aperture Z-scan measurement results of ZL-22 and ZL-61 compounds are shown in Figure 2 and Figure 3, respectively. We performed intensity-dependent Z-scan measurements at each excitation wavelength. The Z-scan results clearly demonstrate that these two compounds both have a strong reverse saturation absorption (RSA) effect at all excitation wavelengths. The third-order and fifth-order nonlinear optical coefficients (β and γ) are used to fit the experimental data [15], and the fitting results are summarized in Table 2.

Based on the Z-scan measurements, the two-photon absorption(TPA) cross sections of ZL-22 and ZL-61 compounds can be determined by the following equation [10]:

(1) $$\sigma_{TPA}=\frac{\hbar \upsilon \beta }{N_{c}}$$

where ℏ is the reduced Planck constant and υ is the frequency of incident light, and $N_{c}\;$ represents the number of molecules per unit volume. The TPA cross sections of our compounds and other BODIPY derivatives are summarized in Table 3. It is shown that both compounds have excellent TPA cross sections in the near-infrared regime, while the nonlinear absorption response of ZL-61 is enhanced compared to ZL-22.

**Figure 4:**
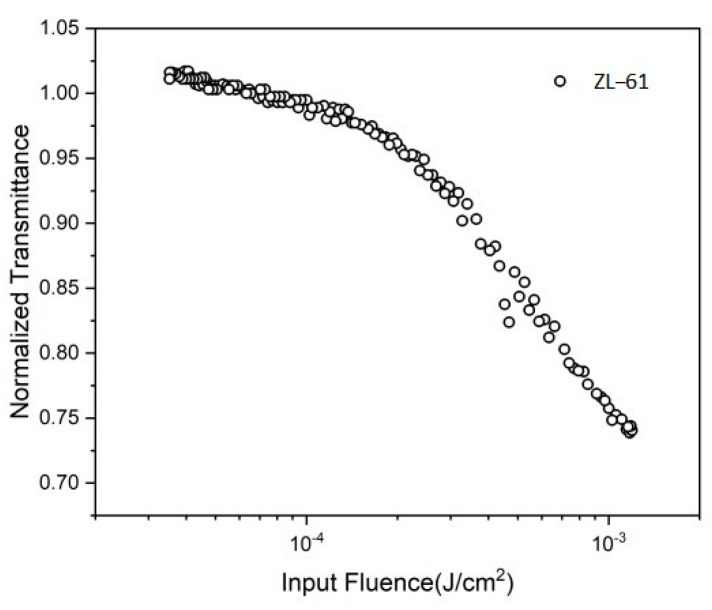
Femtosecond OL of ZL-61 under 800 nm. The dots represent experimental data.

As a basis of the results of Z-scan measurements, we also figured out the femtosecond optical limiting of ZL-61, which represented a better RSA response in the previous testing. The result is shown in Figure 4.

From the figure, we intuitively found that the compound, ZL-61, has an optical limiting response for its decreased normalized transmittance with increased input fluence, which represents a better OL response among the wavelengths of 800~900 nm.

### 3.3. Transient Absorption Spectra and Degenerate Pump-Probe Experiment

**Figure 5:**
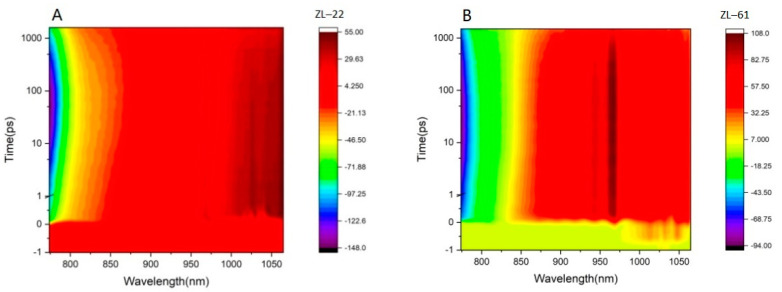
(**A**,**B**) 2-D contour plot of transient absorption as a function of probe wavelength and delay time for ZL-22 and ZL-61, respectively.

**Figure 6:**
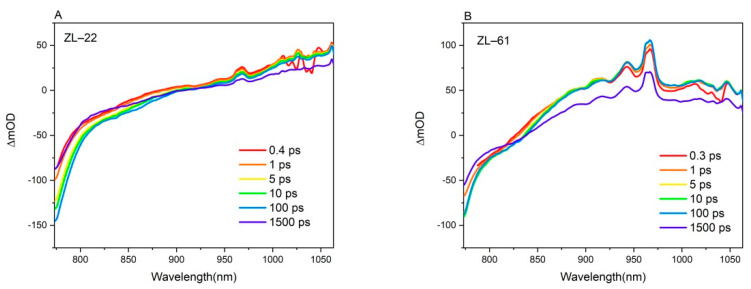
*(***A**,**B**) Femtosecond TA spectra recorded at different delay times of ZL-22 and ZL-61, respectively.

To gain deeper insight into the ultrafast nonlinear optical response of ZL-61 and ZL-22 in solution, femtosecond transient absorption measurements were carried out. Figure 5 and Figure 6 show the two-dimensional (2D) contour plot and the selected curves of TA spectra for ZL-22 and ZL-61 under 650 nm excitation, respectively. A negative peak below 750 nm is observed in ZL-22 after the excitation, which is attributed to the sum effect of ground-state bleaching and stimulated emission. A broad featureless photo-induced absorption (PIA) band is found in the NIR regime (900–1050 nm), which corresponds to the excited-state absorption (ESA) of aza-BODIPY in ZL-22. As for ZL-61, a similar negative peak around 750 nm can also be observed in toluene. Interestingly, a strong PIA band with a positive peak around 960 nm is found in the NIR regime of ZL-61. This absorption feature in the NIR regime was also found in other BODIPY derivatives, which are assigned to the singlet–singlet transition of an excited state [19,20]. Therefore, our TA spectra clearly demonstrate that the excited state of aza-BODIPY is modified by the different donor groups.

As shown in transient absorption spectra, it is clear that all strong excited-state absorptions were present on the charts in the range of 850 to 1060 nm. Moreover, the maximum ESA peaks of the two compounds are similar. The absorption peak of ZL-22 is around 1040 nm, and the absorption peak of ZL-61 is around 960 nm. In the 800 and 850 nm wavelengths, TA spectra can be consistent with the previous Z-scan technique. However, there is one thing that attracts our attention; the charts present saturate absorption of ESA instead of RSA in the range of 800 to 850 nm, for ZL-22; and for ZL-61, when the wavelength is shorter than 800 nm, the value of ∆mOD is less than zero, which indicates saturation absorption. This phenomenon is contrary to the results of Z-scan experiments. This suggests that the RSA effect produced in this waveband is not caused by excited-state absorption, but rather is the result of the combined effect of RSA introduced by TPA and the saturated absorption on an excited state.

**Figure 7:**
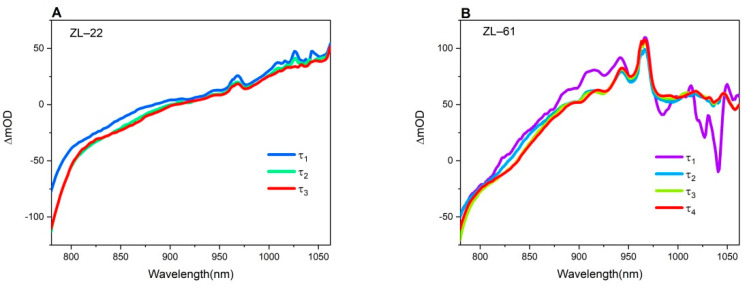
(**A**) Evolution associated difference spectra (EADS) of ZL-22, where $\tau_{1}$ = 4 ps,$\;\tau_{2}$ = 37 ps,$\;\tau_{3}$ = 3565 ps; (**B**) EADS of ZL-61, where $\tau_{1}$ = 0.25 ps, $\tau_{2}$ = 3.5 ps,$\;\tau_{3}$ = 131 ps,$\;\tau_{4}$ = 3311 ps.

To analyze the TA results of the two samples, we used a global analysis of our TA results. The entire TA datasets at all times after the pulse rise are simultaneously fitted to obtain the time constants related to each relaxation process [21,22]. Global analysis results indicate that the TA spectra of ZL-22 in Figure 7 consist of three kinetic components with different time constants, while the TA spectra of ZL-61 consist of four kinetic components.

**Figure 8:**
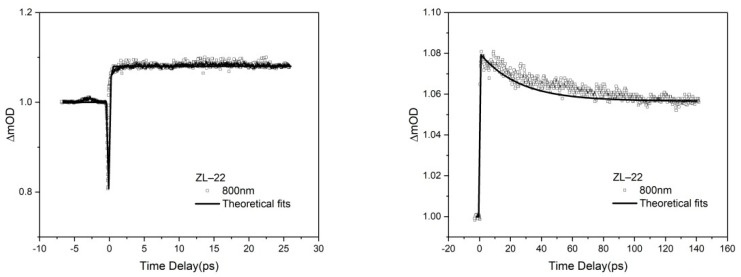
Photodynamic curves of ZL-22 in short-delay time and long-delay time at 800 nm.

**Figure 9:**
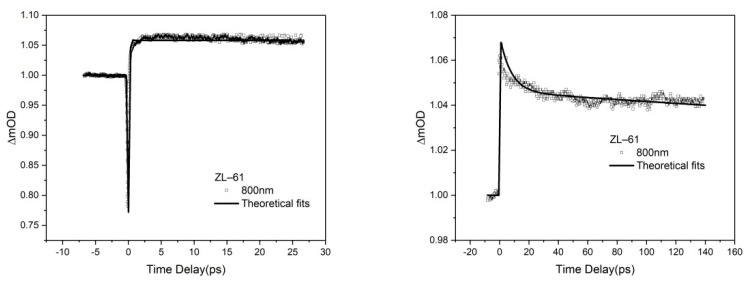
Photodynamic curves of ZL-61 in short-delay time and long-delay time at 800 nm.

In order to verify the strong TPA effect at 800 nm wavelength, we conduct the degenerate pump-probe experiment with pump and probe light both set of 800 nm. We divided the results into long-delay time and short-delay time (Figure 8 and Figure 9) to analyze the ultrafast dynamics. From each short-delay time chart of samples, we observe an ultrafast decrease in normalized transmittance at zero time with a swift recovery in each sample, forming a valley whose duration time corresponds to the pulse width of the pump light. This ultrafast absorption process demonstrates that both the two samples have strong TPA. One can also notice that the transmittance valley of ZL-61 at zero time is deeper compared to that of ZL-22, which indicates that the TPA of ZL-61′s TPA is stronger. Meanwhile, following the ultrafast recovery of the TPA process, the transmittance of both ZL-61 and ZL-22 rise up to NT > 1.05, demonstrating a fast switching from TPA to saturated absorption. In addition, one can find that the saturated absorption in an excited state in ZL-22 is stronger than that in ZL-61. After reaching the transmittance top, both curves descend with relatively long lifetimes, while ZL-61 experiences a faster relaxing than ZL-22, which may result from a shorter singlet excited-state lifetime in ZL-61.

The ultrafast processes revealed in the degenerate pump-probe experiment further explain the optical nonlinearities of TPA and saturated absorption in both compounds. The compounds experience several decay processes related to different excited states after being excited. The following simplified rate equations are built based on effective states for the theoretical analysis of the excited-state dynamics:

(2) $$\frac{dN_{0}}{dt}=-\frac{\sigma_{0}I_{e}N_{0}}{\hbar \omega }-\frac{\beta I_{1}^{2}}{2\hbar \omega }+\frac{N_{1}}{\tau_{1}}+\frac{N_{3}}{\tau_{3}}$$

(3) $$\frac{dN_{1}}{dt}=-\frac{\sigma_{1}I_{e}N_{1}}{\hbar \omega }+\frac{\sigma_{0}I_{e}N_{0}}{\hbar \omega }-\frac{N_{1}}{\tau_{1}}-\frac{N_{1}}{\tau_{ISC}}+\frac{N_{2}}{\tau_{2}}$$

(4) $$\frac{dN_{2}}{dt}=\frac{\sigma_{1}I_{e}N_{1}}{\hbar \omega }+\frac{\beta I_{1}^{2}}{2\hbar \omega }-\frac{N_{2}}{\tau_{2}}$$

(5) $$\frac{dN_{3}}{dt}=-\frac{\sigma_{2}I_{e}N_{3}}{\hbar \omega }-\frac{N_{3}}{\tau_{3}}+\frac{N_{1}}{\tau_{ISC}}+\frac{N_{4}}{\tau_{4}}$$

(6) $$\frac{dN_{4}}{dt}=\frac{\sigma_{2}I_{e}N_{3}}{\hbar \omega }-\frac{N_{4}}{\tau_{4}}$$

with $N_{n}$ representing the density of the number of particles, $\sigma_{n}$ meaning the absorptive cross section.$\;I_{e}$ representing pump intensity, and $\tau_{n}$ standing for lifetimes of each effective state. For ZL-22, $\tau_{1}$ = 0.07 ns, $\tau_{2}$ = 50 fs, $\tau_{3}$ = 45 ns, $\tau_{4}$ = 100 fs, and $\tau_{ISC}$ = 0.04 ns. For ZL-61, $\tau_{1}$ = 0.01 ns, $\tau_{2}$ = 30 fs, $\tau_{3}$ = 1 ns, $\tau_{4}$ = 25 fs, and $\tau_{ISC}$ = 0.035 ns. From the fitting results, the excited-state absorption cross section ($\sigma_{1}$) of both compounds is smaller than their ground-state absorption cross section ($\sigma_{0}$) at 800 nm (ratio of $\sigma_{1}$ to $\sigma_{0}$ is 0.66 for ZL-61 and 0.29 for ZL-22), which correspond to the results shown in Figure 7 and Figure 8.

### 3.4. Transient Absorption Spectra and Degenerate Pump-Probe Experiment

**Figure 10:**
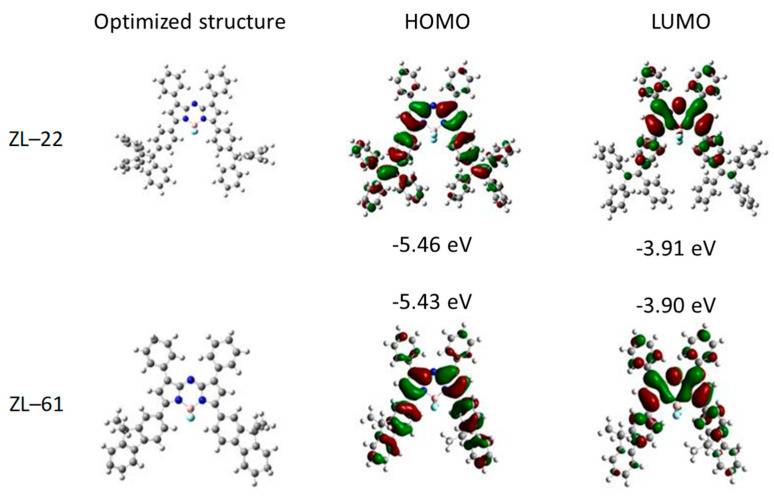
Optimized structures and frontier orbital distributions of ZL-22 and ZL-61 [14].

To gain more insight into the photophysical properties of ZL-61 and ZL-22, the quantum chemical calculations of these two compounds [14] are performed and illustrated in Figure 10. The optimized geometries show that ZL-22 possesses a worse planar conformation than that of ZL-61, due to a larger torsional angle between the BODIPY unit and tetraphenylethylene groups originating from the steric hindrance, and the unique torsional effect of tetraphenylethylene groups in ZL-22 [23]. The calculated highest occupied orbital (HOMO) and the lowest unoccupied orbital (LUMO) levels of these two compounds are shown in Figure 10. The distribution of frontier orbital reveals that these two compounds exhibit a π-conjugation system, which is desirable for the NLO effect. The HOMO of these two compounds shares a similar distribution and is delocalized on the whole molecular skeleton. However, there is little difference in LUMO distribution between the two compounds. The LUMO of ZL-22 is mainly located on the BODIPY unit and extends to the adjacent benzene ring in each of the tetraphenylethylene groups. However, the LUMO of ZL-61 is delocalized on the overall molecular skeleton, though there is a decrease in electronic cloud density of the modified group (fluorene unit) in comparison with HOMO. This observation may explain the tiny difference between the band gaps in both compounds, resulting from the exhibition of the torsional effect. The band gaps are 1.55 eV for ZL-22 and 1.53 eV for ZL-61, respectively, which agree with the value from their UV–Vis experiment. Together with the results of the Z-scan, ZL-61 possesses stronger optical nonlinearities, suggesting that force planar conformation may be more beneficial to NLO absorption properties.

## 4. Conclusions

The nonlinear absorption properties and ultrafast excited-state dynamics of two BODIPY compounds, ZL-22 and ZL-61, were investigated by femtosecond Z-scan experiments and ultrafast pump-probe measurement. The Z-scan results show that both compounds have excellent TPA cross sections in the near-infrared regime, while the TPA cross section of the BODIPY core is enhanced with the increase in the strength of electron-donating groups. The changes in structure caused improvements in photophysical properties, which are confirmed by femtosecond pump-probe measurement and DFT calculations. Our comprehensive investigations of the nonlinear optical response of BODIPY derivatives suggest that the ZL-61 is a promising candidate for two-photon-induced fluorescence and optical limiting applications.

## Figures and Tables

**Table 1:** Absorption spectrum properties of experimental dyes [14].

Dyes	$\lambda_{\mathrm{abs}}{\;}^{a}/\mathrm{nm}$	$\varepsilon /M^{-1}{\mathrm{cm}}^{-1}$	$\lambda_{\mathrm{em}}{\;}^{b}/\mathrm{nm}$	Stokes Shift/nm	$\Phi {\;}^{c}$
ZL-22	708	77500	754	46	0.45
ZL-61	708	92400	742	34	0.42

^a^ In toluene ($c=4\times 10^{-6}\;M$. ^b^ In toluene ($c=4\times 10^{-7}$ M, $\lambda_{ex}=$ 520 nm). ^c^ Fluorescence quantum yields determined by using zinc phthalocyanine (Φ= 0.30 in N,N-dimethylformamide) as standard.

**Table 2:** NLO Parameters of ZL-61 and ZL-22 determined by the femtosecond Z-scan experiments. β represents the two-photon absorption coefficient, and γ represents the effective three-photon absorption coefficient.

Wavelength (nm)	Samples	Nonlinear Coefficient	
		$\beta \;(\times 10^{-12}m/W)$	$\gamma \;(\times 10^{-27}m^{3}/W^{2})$
**800**	ZL-61	5.4	3.9
	ZL-22	0.88	3.8
**850**	ZL-61	2.1	2
	ZL-22	1.2	2

**Table 3:** Summary of the TPA cross sections of ZL-61, ZL-62, and other representative BODIPY derivatives in the near-infrared regime.

Materials	Wavelength λ(nm)	Absorption Peak (nm)	Pulse Width	$\sigma_{TPA}(\mathrm{GM})$
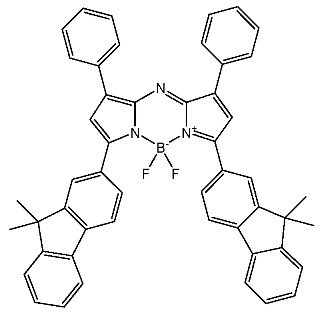 **ZL–61**	800	708	190 fs	8321
	850	708	190 fs	3045
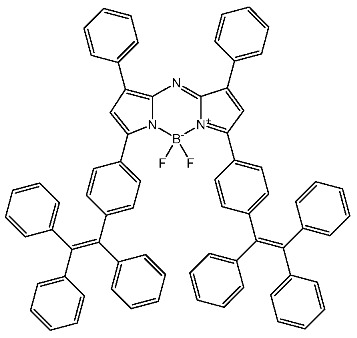 **ZL–22**	800	708	190 fs	1864
	850	708	190 fs	2398
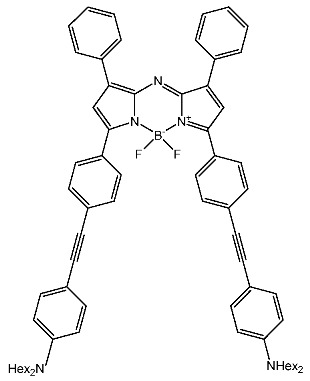 **B^3^ [16]**	1250	745	130 fs	1070 ± 100
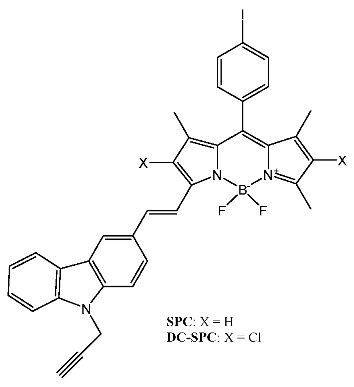 **SPC(in chloroform) [17]**	760/800	588	200 fs	700
**DC-SPC(in chloroform) [17]**	840/800	619	200 fs	800
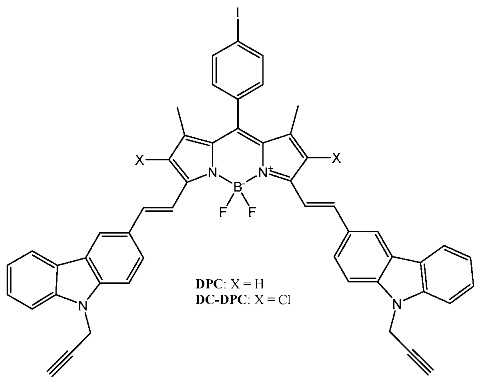 **DPC(in chloroform) [17]**	840/800	674	200 fs	1200
**DC-DPC(in chloroform) [17]**	980/800	703	200 fs	250
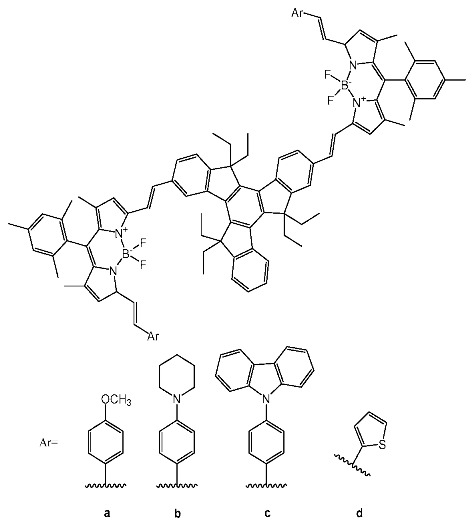 **8a–d [9]**	800	659–677	180 ps	>5000
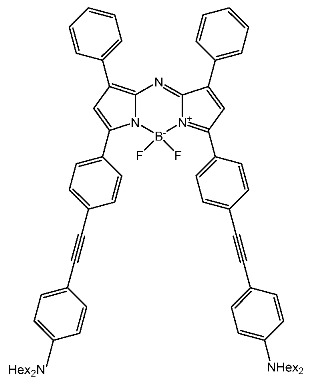 **1(in dichloromethane) [16,18]**	1550	740	150 fs	236
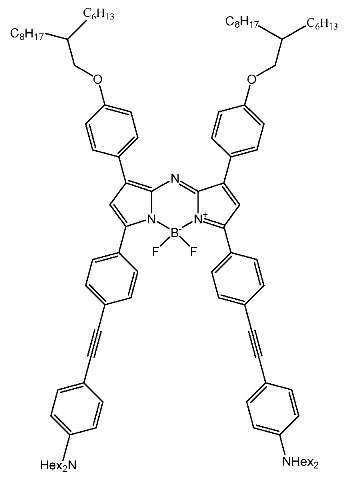 **3(in dichloromethane) [18]**	1550	736	150 fs	320 ± 50

## Data Availability

Not applicable.

## Sample Availability

Samples of the compounds are not available from the authors.
